# Early Postnatal Care Utilization among Rural Women in Horo Guduru Wollega Zone, Ethiopia

**DOI:** 10.4314/ejhs.v32i3.14

**Published:** 2022-05

**Authors:** Lalisa Ayele Woldasemayat, Abiru Neme Negawo, Chaluma Kumela Mengesha, Tilahun Fufa Debela

**Affiliations:** 1 MSc in Maternity Health Nursing, School of Midwifery, Faculty of Health Science, Institute of Health, Jimma University, Jimma, Ethiopia; 2 Msc in Adult Health Nursing, School of Nursing, Faculty of Health Science, Institute of Health, Jimma University, Jimma, Ethiopia; 3 MSc in Health Informatics, Directorate of Policy, Planning, Monitoring and Evaluation, Federal Ministry of Health-Ethiopia/Embedded by JSI/L10K, Addis Ababa, Ethiopia; 4 MPH in Health Service Management, Department of Health Service, Management and Policy, Faculty of Public Health, Institute of Health, Jimma University, Jimma, Ethiopia

**Keywords:** Postnatal, Utilization, Rural, Horo Guduru Wollega, Ethiopia

## Abstract

**Background:**

Postnatal care is a key strategy to reduce maternal mortality. An early postnatal visit is a critical time for the survival of mothers and newborns. Despite the benefits, most mothers do not receive postnatal care services. Thus, this study was aimed to assess early postnatal care utilization among rural women and identify its associated factors.

**Methods:**

Community-based cross-sectional study was conducted in the Horo Guduru Wollega zone from May 10 to 27/2019. A total of 695 randomly selected women participated in the study. A simple random sampling method was employed using the women's registration logbook. Multivariate logistic regression was used to control for possible confounders. A significance level of less than 0.05 was used in the final model to judge statistical significance.

**Results:**

The magnitude of early postnatal care utilization was 21.8%. Multiple logistic regression analysis revealed that decision-making power, awareness about postnatal care, knowing at least one danger sign, place of delivery (AOR = 8.01), and model household (AOR = 5.65) were statistically significant.

**Conclusion:**

This study showed that the utilization of early postnatal care among rural women was found to be low. Decision-making, awareness about the danger signs, place of delivery, and graduating as a model household were the factors associated with postnatal care. Therefore, recommended that health facilities should work on increasing community awareness about the danger signs that can occur after birth or during the postnatal period and increase institutional deliveries.

## Introduction

In the world, women suffer and die from serious health conditions during pregnancy and childbirth(1). Every day in 2017, about 810 women died due to pregnancy and childbirth-related causes. The majority (94%) of all maternal deaths occurred in low and middle-income countries, with almost two-thirds (84%) occurring in sub-Saharan Africa and southern Asia. Sub-Saharan Africa alone accounted for approximately two-thirds of maternal deaths, while population of 570,040 people; 50.1% of whom are males. There were one town administrative and nine rural districts in the zone.

Study population

The study populations were all randomly selected women who gave birth one year before the study period and who lived in the selected Kebele for at least six months were included in the study. Women with stillbirths were excluded from the study.

The sample size was determined using the single population proportion formula by considering the proportion of postnatal care 37.2%; a study from southern Ethiopia(14), the margin of error 0.05 and 95% confidence interval. Hence, the required sample size was 358. Since the multistage sampling was used, a design effect of 2 was taken. After adding a 5% non-response rate, the final sample size was 752. Five Woredas were selected from nine by a lottery method. From these Woredas 21 Kebeles (lowest administrative level in Ethiopia) were selected by the lottery method. Proportional allocation was done for each Kebele based on estimated live births. To have individual study participants, simple random sampling was employed from the women's registration logbook that was served as a frame.

Data collection tools and measurement

The questionnaire was developed after reviewing different kinds of literature including World Health Organization (WHO) guidelines (3,12–17). The questionnaire consists of sociodemographic characteristics, awareness, and attitudes, health system, and obstetric-related variables. The outcome variable was assessed based on the binary outcome, with yes or no responses. A woman is considered as utilized if she gets early postnatal care service at least once during the first 7 days after childbirth for reasons relating to postpartum care. The attitude of women towards early postnatal was measured by asking four closed-ended questions with positive, neutral, and negative responses. Those women, who agreed/very agreed or answer positively, were considered as a positive attitude and those respondents very disagreed/disagreed or negatively responded were considered negative attitude. Those women who responded as neutral were also considered medium.

Data were cleaned and entered into EpiData version 3.1 and exported to SPSS version 20 for analysis. Both descriptive and inferential statistics were done. Descriptive analysis was done for socio-demographic variables. Bivariate logistic regression analysis was done to identify a candidate for multivariate analysis. Variables with a p-value of <0.25 were included in multivariable logistic regression analysis. Lastly, variables with a p-value of <0.05 in the final regression were considered as statistically significant associated with the outcome variable.

Data quality management

To ensure the quality of data, a questionnaire was translated to the local language, training was given for data collectors and supervisors, a pre-test was done on 35 (5%) of the total sample size, and a necessary adjustment was made before used for actual data collection.

## Results

Socio-demographic Characteristics

Out of 752 women, 695 participated in the study yielding a response rate of 93%. Two hundred five (29.6%) of the respondents were in the age of 25–29 with the mean (+SD) age of 26. 98 (+ 5.34) years. Six hundred forty-six (93%) of study participants were married. The dominant ethnic group was Oromo (94%). Two hundred ninety-two (42%) and 250(36%) of the respondents were followers of Protestant and Orthodox religions respectively (Table 1).

**Table 1:** Socio-demographic characteristics of study participants in Horo Guduru Wollega Zone, 2019(n=695)

Variables	Categories	Frequency	%
Age of mother	15–19	120	17.2
	20–24	168	24.2
	25–29	206	29.6
	30–34	130	18.8
	>=35	71	10.2
Marital status	Married	646	93
	Divorced	26	3.8
	Single	23	3.2
Ethnicity	Oromo	653	94
	Amhara	35	5
	Tigre	7	1
Religion	Protestant	292	42
	Orthodox	250	36
	Muslim	153	22
The education level of mothers	Unable to read and write	247	35.5
	Primary education	325	46.8
	Secondary education and above	123	17.7
The education level of the father	Unable to read and write	167	24.1
	Primary education	311	44.7
	Secondary education and above	217	31.2
Occupation of Mother	Housewife	632	90.9
	Merchant	56	8.1
	Others*	7	1
Occupation of father	Farmer	530	76.3
	Merchant	64	9.1
	Government employee	71	10.2
	Others**	30	4.3
Wealth status	Richest	101	14.5
	Rich	108	15.6
	Middle	142	20.4
	Poor	157	22.6
	Poorest	187	26.9

* Others: Daily laborer and student

** others: Carpenter and government workers

**Figure 1:**
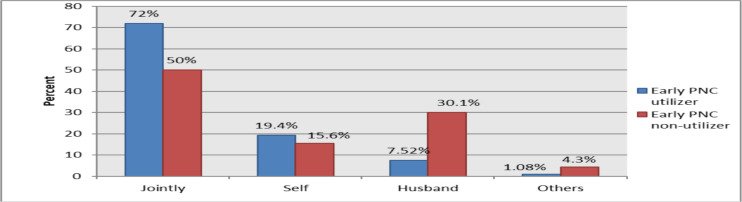
Bar graph depicting the power of decision making of mothers on the matter of early postnatal care (PNC) utilization in Horo Guduru Wollega zone, 2019(PNC users= 152).

**The decision-making power**: Of the respondents, 152 (21.8%) utilized early postnatal care services. Among women who utilized the early postnatal care, the majority (72%) of them decide jointly with their husbands while nearly one-fifth (19%) of them decide by women themselves. Of women who do not utilize early postnatal care half (50%) of them decide jointly with their husband while about one-third (30%) of them the decision to utilize health services was made by their husbands (Figure 1).

**Awareness and attitude of women**: Among the study participants, 314(45.2%) of study participants had ever heard about early postnatal care services while about two-thirds (66.1%) of respondents know at least one danger sign. Four hundred fifty-six (65.6%) of study participants know the availability of early postnatal service. Three fourth (75.3%) of the study, participants have a positive attitude towards the utilization of early postnatal care (Table 2).

**Table 2:** Awareness and attitude related factors among rural mothers in Horo Guduru Wollega Zone, 2019(n=695)

Variables	Categories	No	%
Ever heard about early postnatal care service	Yes	314	45.2
	No	381	54.8
Know at least one danger sign	Yes	459	66.1
	No	236	33.9
Know the availability of postnatal care service	Yes	456	65.6
	No	239	34.4
Attitude towards early postnatal care services	Negative	77	14
	Moderate	60	10.8
	Positive	415	75.3

Health system-related characteristics: Four hundred seventy-one (67.7%) of women had given birth at home. The majority (84.4%) of study participants were graduated as a model family (the training given by health extension workers on basic health care). Five hundred seventy-five (82.8%) of study participants reach the nearest health facility in less than one hour (Table 3).

**Table 3:** Health system-related characteristics among rural mothers in Horo Guduru Wollega Zone, 2019(n=695)

Variables	Categories	Frequency	%
Place of delivery	Home	471	67.7
	Health facility	224	32.3
Model Household	Not graduated	108	15.6
	Graduated	587	84.4
Distance to a health facility	<1 hour	575	82.8
	>=1hours	120	17.2

**Obstetric characteristics**: From the respondents, 220(31.7%) of study participants had no antenatal care visit while 198(28.5%) had four times or more follow-up. The majority (87.1%) had no history of abortion. The mode of delivery for almost all (95.7%) of study participants was spontaneous vaginal delivery. More than half (57.5%) of women had desired pregnancy (Table 4).

**Table 4:** Obstetrics characteristics of rural mothers in Horo Guduru Wollega zone, 2019(n=695)

Variables	Categories	Frequency	%
ANC follow-up	No visit	220	31.7
	Once	60	8.6
	Twice	71	10.2
	Three times	146	21
	>=4 times	198	28.5
Abortion history	No	605	87.1
	Yes	90	12.9
Mode of delivery	Spontaneous vaginal delivery	665	95.7
	Forceps delivery or cesarean section	30	4.3
Desire to pregnancy	Wanted no more	149	21.5
	Wanted later	146	21
	Wanted then	400	57.5

**Factors association with postnatal care**: Among the variables, the decision-making power of women, awareness about postnatal service and knowing at least one danger sign, place of delivery, and graduating as a model household were significantly associated with early postnatal care utilization. By keeping other variables constant, women who make the decision jointly with their husband had 9.34 (AOR = 9.34; 95% CI: 3.18, 27.39) times more likely to utilize early postnatal care as compared to women who decide by themselves. Women who had ever heard about the early postnatal service were 5.25 (AOR = 5.25; 95% CI: (2.09, 13.19) times more likely to utilize early postnatal care as compared to women who had no information about the services. Again, by adjusting other variables constant, women who knew at least one danger sign of not utilizing early postnatal care were 3.41 (AOR = 3.41; 95% CI: 1.80, 6.39) times more likely to utilize early postnatal care as compared to women who did not know the danger signs.

Place of delivery was also another factor that associates with the utilization of early postnatal care. Women who gave birth at the health facility were 8.01(AOR = 8.01; 95% CI: 4.23, 15.20) times more likely to utilize early postnatal care as compared to women who gave birth at their home. Similarly, by keeping other variables constant, women from the model households who graduated after training on basic health extension program had 5.65 (AOR = 5.65; 95% CI: 2.84, 11.23) times more likely to utilize early postnatal care as compared to those women who were from non-model households (Table 5).

**Table 5:** Factors associated with postnatal care service among mothers in Horo Guduru Wollega zone, 2019

Variables	Categories	Utilized No (%)	Non- utilized No (%)	COR (95%CI)	AOR (95%CI)
Decision maker	Women herself(a)	29(19.4)	85 (15.6)	1	1
	Her husband	109(72)	272(50)	.86 (.49, 1.50)	.92 (.42, 2.01)
	Jointly with husband	12(7.5)	163(30.1)	4.96 (2.31, 10.65)	9.34 (3.18, 27.39) *
	Others	2(1.1)	23(4.3)	4.96(.98, 25.21)	3.21 (.45, 23.10)
Ever heard about early postnatal care service	Yes	142(45.2)	49(9)	8.19 (4.60, 14.57)	5.25 (2.09,13.19) *
	No(a)	172(54.8)	494(91)	1	1
Knows at least one danger sign	Yes	100(66.1)	146(26.9)	5.31 (3.41, 8.28)	3.41 (1.80, 6.39) *
	No(a)	52(33.9)	397(73.1)	1	1
Place of delivery	Health institution	103(67.7)	129(23.7)	6.75 (4.29, 10.70)	8.013 (4.23, 15.20) *
	Home(a)	49(32.3)	414(76.3)	1	1
Model Household	Yes	128(84.4)	283(52.2)	4.96 (3.04, 8.10)	5.65 (2.84, 11.23) *
	No(a)	24(15.6)	259(47.8)	1	1

* P<0.05

(a) reference category

## Discussion

In this study, the magnitude of early postnatal care utilization was 21.8% (95%CI: 18.96%–24.04%). Our finding was in line with a study conducted in the Asako district of Ethiopia, in which the magnitude of early postnatal care utilization was 23.7% (21). But this result is lower than other studies done in the country. Studies done in Debra Markos (20), Mekelle (16), Southern Ethiopia(22), and North Showa of Ethiopia (23) indicated its utilization was33.5%, 32.2%, and 28.4% respectively. The possible explanation for the difference might be due to the study area. Our study was exclusively done in rural areas. This study finding is also lower than the study done in Adigrat town, Tigrai region of Ethiopia. The result of early postnatal care utilization in Adigrat was 34.3% (13). The possible explanation for the difference was might be due to the study area which was done among town dwellers.

Keeping other variables constant, women who make the decision jointly with their husbands had 9.34 times more likely to use early postnatal care as compared to women who decide by themselves. This result is in line with the theory of joint decision, helps to bring together available information, reconcile objectives and then make an effective decision together (24,25). Areas in which women and their husbands make the decision jointly and areas where women decide by themselves utilization of early postnatal care are higher. In the areas where a decision is made only by a husband and other families, it is lower. The result is also in line with a study done in the Aseko district, Arsi zone of Ethiopia (21).

Women who had ever heard about the service were 5.25 times more likely to utilize postnatal care than women who had no information about the services. This study is in line with a study conducted by Debra Berhan and Shebe Sombo in which the early postnatal service has been affected by the awareness about service (26,27). Having information about one service facilitates its utilization(28). A woman who knew at least one danger sign of obstetric complication after birth had 3.41 times more likely to utilize early postnatal care as compared to those who did not know. The result was also supported by other studies done in Debra Birhan and Halaba of Ethiopia in which the likeliness of utilizing postnatal service greater among women who knew danger signs (18,27).

Our study revealed that woman who gave birth at the health institution was 8.01 times more likely to utilize early postnatal care than women who gave birth at home. The result was in line with other studies in the country and other parts of the world (15,20,27,29). During the institutional delivery women and her family get additional information about the health of the mother and newborn which may lead to use the service. Again, our result is supported by a study done in the Aseko district; in which mothers who gave birth in the health institutions are more likely to utilize early postnatal care services(21).

The result of the current study points out that, women who were from the model households had 5.65 times more likely to utilize early postnatal care than those women from nonmodel households. Model household is the approach by which household members get training on health extension programs in Ethiopia (30,31). The aim is to prevent disease and promote health to ensure the utilization of health services by enabling households to implement most health interventions. Households are graduate after fulfilling 75% of the health extension package (30). This might be the possible explanation why women from graduated households use postnatal care services more.

The study used a standard tool to measure the early postnatal care services. However, this study was not out limitation: although used standard tool for measurement and participants were assured confidentiality, the equal chance was not given for all women in the zone and nature of the cross-sectional study in which the timing snapshot is not guaranteed to be representative. Not with standing this limitation, we believe that our study has very important findings for strengthening the early postnatal care services for current health service deliveries in the study area and areas with similar settings.

In conclusion, our findings revealed that early postnatal care utilization in rural areas was found to be low. The utilization of early postnatal care was influenced by the decisionmaking power of women, awareness related to postnatal service, knowledge of danger signs, attending a birth at a health institution, and graduating as a model household. Therefore, it is recommended that health facilities should work on increasing the community's awareness about the danger signs that can occur after birth or during the early postnatal period. Moreover, the concerned body should work on women's empowerment, since the decision-making power plays an important role in the utilization of early postnatal care.
